# SSViT-YOLOv11: fusing lightweight YOLO & ViT for coffee fruit maturity detection

**DOI:** 10.3389/fpls.2025.1691643

**Published:** 2025-12-01

**Authors:** Yifan Liu, Qiudong Yu, Shuze Geng, Shiyi Guo, Ling Liu

**Affiliations:** 1 College of Information Technology engineering, Tianjin University of Technology and Education, Tianjin, China; 2 College of Computer Science and Information Engineering, Tianjin Agricultural University, Tianjin, China

**Keywords:** object detection, coffee fruit maturity, YOLOv11, vision transformer, lightweight

## Abstract

Accurate identification of coffee fruit maturity is critical for optimizing harvest timing and ensuring bean quality, but manual inspection is time-consuming and prone to subjectivity. Automated visual detection faces challenges including subtle color differences among maturity stages, frequent occlusions within fruit clusters, variable lighting, and abundant small-scale targets. In this paper, we propose SSViT-YOLOv11, an improved YOLOv11n-based framework that integrates Single Scale Vision Transformer (SSViT) into the backbone and refines multi-scale feature fusion to enhance context modeling and small-object representation. The C3K2 modules in YOLOv11n are integrated with Arbitrary Kernel Convolution (AKConv) and multi-scale convolutional attention (MSCA) is added in the head, effectively improving detection accuracy and rendering the model more lightweight. Experimental results show that SSViT-YOLOv11 achieves superior performance across multiple evaluation metrics. Specifically, the model attains a precision of 81.1%, a recall of 77.4%, and a mean Average Precision (mAP@50) of 84.54%, while operating at 23 FPS and requiring only 2.16 million parameters. These results indicate that the proposed model offers a favorable balance of accuracy, inference speed, and model compactness, making it well suited for assisting farmers in coffee fruit maturity assessment.

## 1 Introduction

Coffee is the second most consumed commodity in the world after oil, and its quality directly affects the drinking experience of billions of consumers and the economic benefits of the global coffee industry (Honda et al., 2024). The maturity of coffee fruit is one of the key factors determining the quality of coffee. The traditional method for determining the maturity of coffee fruits mainly relies on manual experience. This manual approach is inefficient and highly subjective, hindering the consistency and accuracy of maturity assessment results.

### 1.1 YOLO-based agricultural detectors

With the rapid development of computer vision and deep learning, image-based object detection technology provides new solutions for agricultural intelligence (Feng et al., 2023; Hussain, 2024; Zhang et al., 2024a, 2022). Among them, the YOLO object detection algorithm has been widely applied due to its real-time performance and versatility. Badeka et al. proposed an improved YOLOv7 algorithm integrated with an attention mechanism to detect the ripeness of grapes. This algorithm was successfully deployed on automated detection robots, effectively improving detection efficiency and accuracy (Badeka et al., 2023). Francisco Oliveira et al. proposed an improved YOLOv7 of node detection in grapevines, achieving F1-Score values between 70% and 86.5% with inference times of around 89 ms for an input size of 1280×1280 px (Oliveira et al., 2024). Chen et al. proposed an improved multi-task deep convolutional neural network (DCNN) based on YOLOv7 in detection of fruit and fruit-cluster ripeness. The method achieved an overall score of 86.6% in mAP, with an average inference time of 4.9 ms (Chen et al., 2024). Zuo et al. proposed an improved YOLOv8 named LBDC-YOLO (Lightweight Broccoli Detection in Complex Environment-YOLO), incorporated the Slim-neck design paradigm based on GSConv and Triplet Attention in the backbone. Experimental results demonstrate the method achieved an average detection accuracy of 94.44% for broccoli (Zuo et al., 2024). Zhang et al. proposed an improved YOLOv7-tiny with ELAN_Partial Conv, Content-aware ReAssembly of Features (CARAFE) and Coordinate Attention (CA), the model achieved a mAP@0.5 of 94.3% and a frame rate of 29.7 FPS (Zhang et al., 2024b). Shen et al. proposed YOLOv8 for tomato leaf disease detection. The model integrated depth wise Convolutions and SE_Block, achieved precision 85.7%, recall 72.8%, and mAP@0.5 79.8%, mAP@0.5:0.95 51.6% (Shen et al., 2025). Zhu et al. proposed a EfficientDet model for the fruit maturity detection of multi-cultivar olives in orchard environments. The CBAM and Bi-FPN are embedded into the model, achieved more than 93% mAP for the maturity detection of four olive cultivars (Zhu et al., 2024). Zhu et al. proposed an improved YOLOv11n for detection and grading of Olive Ripeness, which replaced the backbone with EfficientNet-B0, integrated the LSKA mechanism and the BiFPN. The mean average precision (mAP) of fruit maturity detection reached 94.60%, 95.45%, 93.75%, and 96.05% for 4 olives of cultivar, the mean detection time was 337 ms per image (Zhu et al., 2025). Xiao et al. proposed an improved YOLOv5 for detection of blueberry fruit maturity. The ShuffleNet and CBAM are embedded into the model, the average recall is 92.0%. The mAP@0.5 is 91.5%, the average speed is 67.1 fps (Xiao et al., 2024). Yang et al. proposed GTDR-YOLOv12. The backbone integrated Ghost Conv., Dynamic ReLU and Task-Dependent Attention Mechanisms, achieved precision 88.0%, recall 83.9%, F1-score 85.9%, mAP:0.5 90.0%, GFLOPs 4.8, and the number of parameters 2.23 M (Yang et al., 2025). Covering works that enhance YOLO variants with attention mechanisms, lightweight backbones (e.g., ShuffleNet, EfficientNet), or neck optimizations (e.g., BiFPN, Triplet Attention). We critically note that while these improve accuracy, many still struggle with small-object sensitivity and occlusion robustness, and often increase model size.

### 1.2 Transformer-based agricultural detectors

Compared with CNNs, Transformers are better able to model global information, whereas the latter mainly focus on extracting local features. This unique advantage has made Transformers quickly become a research hotspot and gradually introduced into the field of Computer Vision. In recent years, the research on applying Transformers and their improved models to agricultural detection has gradually increased, and remarkable achievements have been made in multiple aspects. Wu et al. proposed a two-stage region proposal method using Swin Transformer for few-shot pest detection, which achieved an mAP@50 of 95.71 and an F1 score of 91.03 (Wu et al., 2023). Bai et al. proposed a lightweight pest identification model based on Transformer and super-resolution sampling techniques, which achieved precision, recall, mAP, and FPS of 0.97, 0.95, 0.95, and 57, respectively (Bai et al., 2023). Bo et al. proposed an improved lightweight recognition model for harvested cherry tomatoes. The backbone is replaced with EfficientViT, and an adaptive detail fusion module is designed. Experimental results show that this lightweight model achieves fast detection (41.2 f/s) and low computational load (8.7×10^9^ FLOPs) while maintaining a high recognition accuracy (90%) (Bo et al., 2024). Barman et al. proposed a ViT for categorizing nine different tomato leaf disease classes and a healthy class, achieved precision 0.95, recall 0.91, F1 score 0.93 (Barman et al., 2024). Including Swin Transformer, EfficientViT, and ViT adaptations for pest or disease detection. We highlight their strength in global context modeling but point out their high computational cost and limited suitability for real-time edge deployment—a key requirement in field agriculture.

### 1.3 Coffee-specific maturity detection approaches

In recent years, some progress has also been made in the application of deep learning to the detection of coffee fruit maturity. Bazame et al. proposed a YOLOv4 model for detecting coffee fruits on tree branches (Bazame et al., 2023). At a resolution of 800×800 pixels, this model achieves a mAP of 81% for immature (green) fruits—significantly outperforming both YOLOv3 (78%) and its lightweight variant (77%). However, the mAP falls to 40% under lower resolution (320×320), primarily due to the visual similarity between immature fruits and leaves (Lab* color difference < 8). Moreover, the detection accuracy decreasing by 12% in shaded environments. Eron et al. introduced a YOLOv7-based approach for coffee fruit maturity detection, reporting an mAP@0.5 of 0.904 for a single-class (fruit-only) setting, 0.892 for binary classification (unripe vs. ripe), and 0.605 for multiclass categorization (green, yellow-green, cherry, raisin, dry) (Eron et al., 2024). The model has 36.9 million parameters, necessitating hardware acceleration for deployment on mobile platforms such as drones. Additionally, its multiclass accuracy remains relatively low, and it is sensitive to variations in fruit density, with recall fluctuating by ±7% in high-density scenarios. Tamayo-Monsalve et al. developed a CNN method for coffee maturity classification using multispectral data integrated with an Inception-ResNet-v2 architecture, achieving over 98% accuracy across five maturity stages (Tamayo-Monsalve et al., 2022). Nevertheless, this approach entails high hardware costs, requiring custom multispectral cameras (priced above $15,000) and a controlled lighting environment. Furthermore, the processing time for a single image exceeds 200 ms, which hinders its suitability for real-time applications. In contrast, Ulhaq et al. proposed a method combining multispectral imaging with a Bayesian classifier that operates solely on CPU, achieving an impressive inference speed of 1,100 FPS and compatibility with embedded devices (‘Ulhaq et al., 2024). However, its classification accuracy caps at 91.01%—approximately 7% lower than CNN-based models—and the false detection rate increases by 38% when applied in complex outdoor backgrounds. Most recently, Kazama et al. enhanced YOLOv8n with an RFCAConv module to detect coffee fruits and classify their maturity stages (Kazama et al., 2024). Their model attained an mAP@0.5 of 74.20%, outperforming the standard YOLOv8n by 1.90% with only a minimal increase in computational demand. Summarizing prior CNN, YOLO, and multispectral methods. We identify persistent gaps: low multiclass accuracy under natural lighting, sensitivity to fruit density, and dependency on expensive hardware (e.g., multispectral cameras).

SSViT-YOLOv11 takes the lightweight YOLOv11n as its basic framework. The specific design motivations and optimization details are as follows:

#### 1.3.1 Targeted optimization of AKC3K2

Design Motivation: Coffee fruits often grow in irregular clusters and exhibit minimal color differences between similar maturity stages, while standard 3×3 convolutional kernels struggle to align with their elliptical or occluded boundaries under scale and shape variations—motivating the need for adaptive, lightweight feature extraction. Targeted Improvements:

- Arbitrary Kernel Convolution (AKConv) is integrated into the bottleneck layer of the C3K2 module in YOLOv11n (instead of merely replacing standard convolutions). Through Offset Learning, the spatial offset of each sampling point is predicted to dynamically adjust the convolutional sampling positions—this enables adaptation to the elliptical or irregular contours of coffee fruits.
- AKConv supports linear adjustment of convolution parameters. When integrated into the C3K2 module, the number of parameters of the modified C3K2 module is reduced by 18.3% compared with the original C3K2 module, while maintaining detection accuracy. Additionally, two variants of AKC3K2True/False are designed for the two structures of the C3K2 module.
- The optimal integration strategy was verified by ablation experiments. The results show that the combination of AKC3K2T/F achieves the best performance: compared with the YOLOv11n baseline, mAP@0.5 increases by 3.08%, the number of parameters decreases from 2.62 million to 1.57 million, and the FPS increases from 20 to 26, thus balancing accuracy, lightweighting, and real-time performance.

#### 1.3.2 Targeted optimization of SSViT

Design Motivation: Severe mutual occlusion in dense coffee fruit clusters confuses local CNN features, yet standard Vision Transformers are too computationally expensive for edge deployment—necessitating a lightweight global modeling approach that leverages high-level semantics without multi-scale overhead. Targeted Improvements:

- Only the deepest C5 feature map output by the backbone network (instead of multi-scale features C3/C4/C5) is input into SSViT, as it contains the richest semantic information, which is crucial for detecting subtle maturity differences.
- A single layer ViT encoder (SSVE) is adopted, combined with a three-scale weight-sharing mechanism. This enables cross-scale feature interaction at minimal computational cost (adding only 0.07M parameters), effectively mitigating missed detections caused by intra-cluster occlusion.
- The standard 1D sine-cosine Positional Encoding (PE) is extended to 2D row-column PE. This enhances spatial information representation and avoids position confusion caused by the permutation invariance of Transformers (e.g., distinguishing fruits at different positions in the same cluster).
- Preprocessing of “Batch Normalization (BN) + AKConv” is added to the input end of SSViT, and a residual connection is established with the original C5 feature map. At the output end, the features processed by SSVE are again fused with the input features of 2D PE via a residual connection, thus enhancing feature robustness and information integrity.

#### 1.3.3 Targeted optimization of MSCA-head

Design Motivation: The wide scale variation of coffee fruits—and frequent occlusion of small fruits by leaves or neighboring fruits—demands a detection head capable of simultaneously preserving fine local details and capturing broad contextual cues with minimal computational cost. Targeted Improvements:

- MSCA employs four parallel convolutional kernels of sizes 5, 7, 11, and 21 within the detection head. The large kernels are implemented via separable strip convolutions, reducing computational cost to only one-third of that of standard convolutions. Small kernels (5×5) preserve small fruit edge and local color features, while large kernels capture global context of large fruits or occluded scenarios.
- Attention maps are applied to features via element-wise multiplication, which amplifies responses in critical regions. This operation amplifies responses in fruit regions and weakens interference from the background. It is particularly effective for unripe fruits with colors similar to leaves, reducing the false detection rate.
- The optimal integration position was verified by ablation experiments. The results show that “pre-head integration” achieves better performance: compared with YOLOv11n, the mAP@0.5 increases by 1.98%, the number of parameters increases by 11.8%, and the FPS decreases only from 20 to 19.

Our primary contribution is a lightweight, synergistic YOLOv11n that strategically integrates three modules. Crucially, this is the first work to (1) apply AKConv to agricultural vision tasks, (2) propose a SSViT that operates only on the deepest semantic feature map to minimize overhead, and (3) demonstrate their complementary benefits through ablation studies showing consistent gains in accuracy, speed, and compactness.

## 2 Materials and methods

### 2.1 Experimental dataset building

The coffee fruit maturity dataset comprises two parts: (1) A public dataset from the ciencia-cafeto workspace (URL: https://www.kaggle.com/datasets/cienciacafeto/coffee-fruit-maturity), containing Arabica coffee fruit images across maturity stages under sunny, cloudy, and partially shaded conditions; (2) A self-collected dataset from a Pu’er, Yunnan coffee plantation, captured via smartphones (1.5m height, 0.8–1.2m target distance) during September–November (ripening season), with most images taken on sunny days and some under cloudy/shaded conditions to enhance lighting robustness. Close-up shots of dense clusters ensure sufficient small-object and occluded samples.

Annotations were completed using Jingling Annotation Assistant (open-source, YOLO-format supported) via manual rectangular bounding box marking. The public dataset was manually screened for high-quality samples and re-annotated. To address small dataset size and sample imbalance (e.g., more unripe vs. fewer overripe samples), Ultralytics library augmentation algorithms (random flipping, mosaic, affine transformation, cropping, padding, etc.) were applied to balance the training set (Khanam and Hussain, 2024).

**Figure 1:**
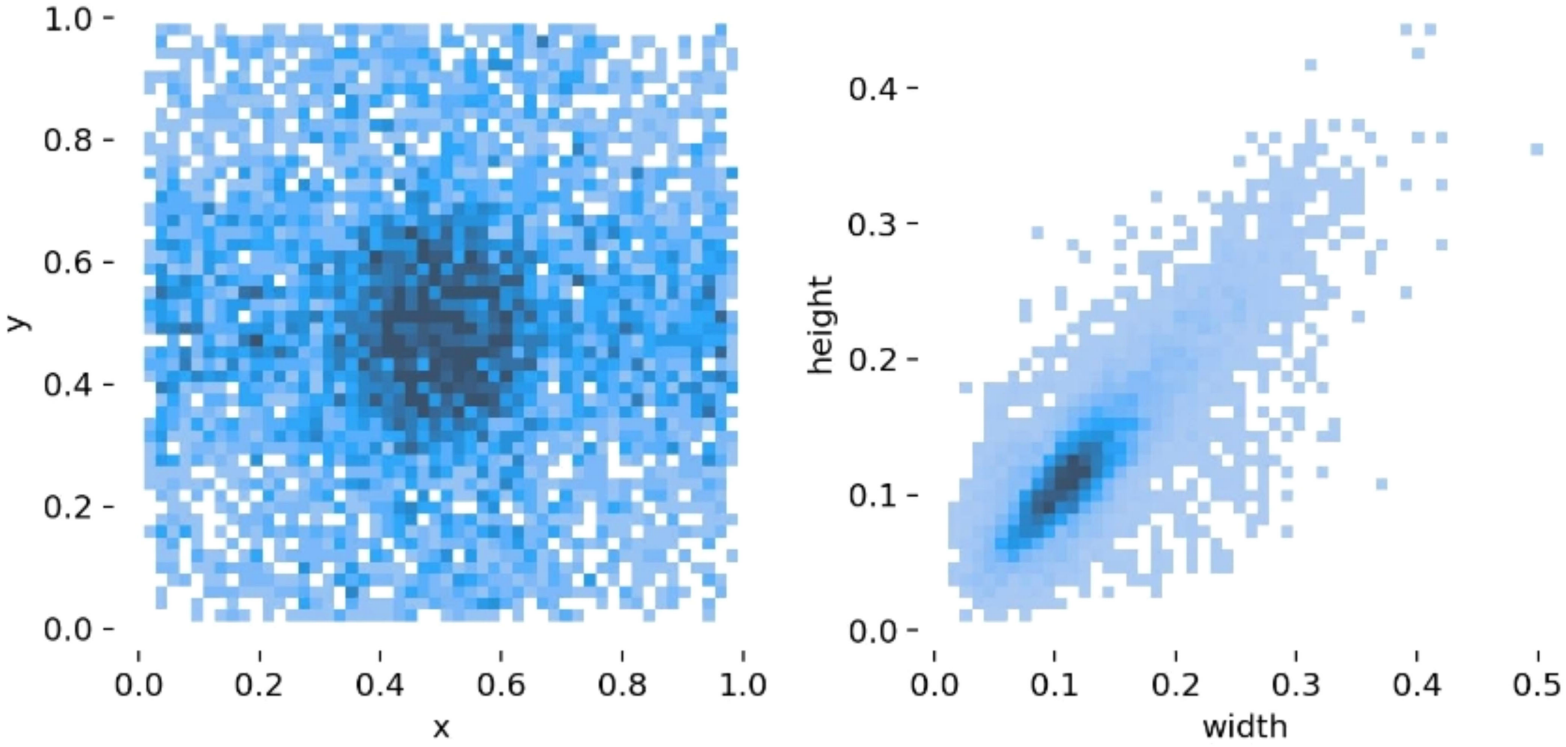
Visualization diagram of dataset bounding boxes.

The number of samples of each category is shown in **Table 1**. Through data augmentation, the dataset was expanded to 800 images. The ratio of training set and valid set is 7:3. **Figure 1** presents a visualization of the detection bounding boxes for this dataset. **Figure 1a** is the spatial distribution map of the bounding boxes. As shown in the figure, the dataset includes cases where the bounding boxes are distributed in most positions of the images. A large number of incomplete fruits, which are at the edges of the images or are incomplete due to occlusion by fruit clusters, have been annotated. The targets in the central part of the whole are relatively concentrated, and most of the edge areas are covered, which meets the actual real-time detection requirements. **Figure 1b** is the width-height scatter density plot. The figure shows that the sizes of the target fruits are mainly distributed in the range of 0.05-0.2 of the total pixel width and height of the images. There are also some targets with larger or smaller sizes than the aforementioned range, which is beneficial for enhancing the generalization ability of the model.

**Table 1:** Number of samples of each maturity type.

Category	Images	Instances
dry	800	726
overripe	800	455
ripe	800	1392
semi_ripe	800	977
unripe	800	6396

### 2.2 Overall architecture of the SSViT-YOLOv11

YOLOv11 is a SOTA object detection model. It is built on the success of previous YOLO versions and introduces new features and improvements to further enhance performance and flexibility. We were motivated by its lightweight design, efficient feature extraction capabilities, optimized architecture including components like C3K2, C2f-RepNCSPFPN, and SPPF modules. YOLOv11n is a lightweight variant derived from YOLOv11, specifically designed to reduce computational resource requirements while maintaining high detection accuracy. All these features contribute to achieving a balance between computational efficiency and detection accuracy that is suitable for real-time deployment in agricultural settings such as coffee orchards.

**Figure 2:**
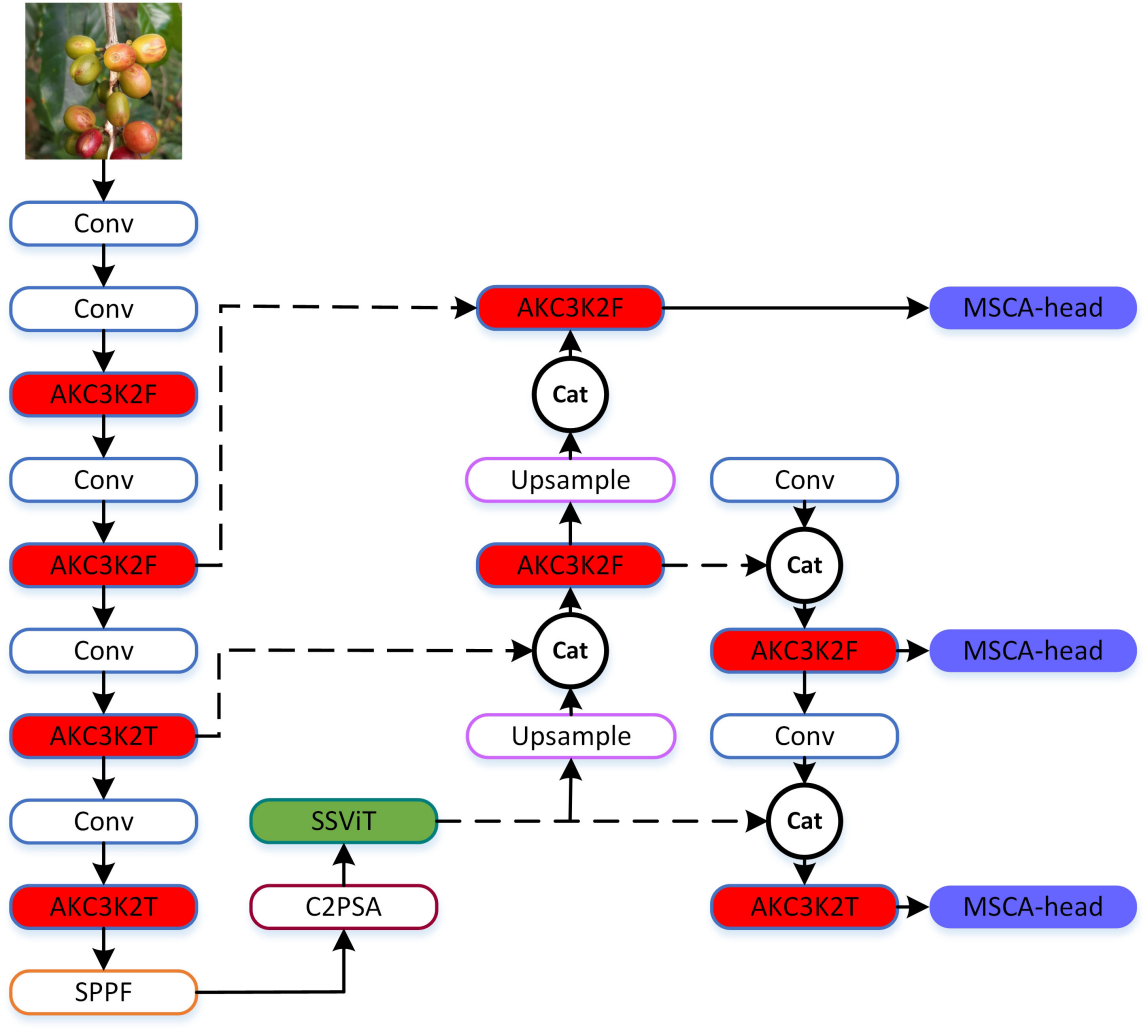
The structure of the SSViT-YOLOv11. AKC3K2F/T correspond to the two structures of AKC3K2 and C3K=True/False in **Figure 4**; MSCA-Head refers to the head architecture shown in **Figure 8a**.

The architecture of SSViT-YOLOv11, which retains the core structure of YOLOv11n while integrating three key lightweight modules: (1) AKC3K2 allows the shape and position of the convolution kernel to be dynamically adjusted according to image content, better matching and covering target regions, and can optimize performance by reducing unnecessary parameters; (2) Single Scale ViT (SSViT) after the deepest feature map to capture global context with minimal computation. This module not only has the advantage of Transformer in global information extraction to improve performance, but also the Single Scale encoder greatly reduces computational complexity, taking into account the real-time detection; and (3) Multi-scale Convolutional Attention (MSCA) in the detection heads to improve multi-scale and occluded object detection. It is a local attention mechanism for multi-scale feature fusion, which can better detect targets of different scales, especially suitable for the situation where coffee fruits are small and grow in clusters. Moreover, the computational complexity is greatly reduced through the combination of strip convolutions. **Figure 2** shows the overall architecture of the SSViT-YOLOv11. The three differently colored filled parts in the figure represent the three improved modules proposed in this paper, with their specific details elaborated in **Sections 2.3 to 2.5**. These modules are strategically placed to address the specific challenges of coffee fruit detection: small targets, occlusion, and embedded deployment constraints.

### 2.3 The structure of AKC3K2

The standard convolutions in YOLOv11n use fixed 3×3 kernels, which are inefficient for irregularly shaped coffee fruits under occlusion and scale variations of the fruits. In this paper, the Arbitrary Kernel Convolution (AKConv) is adopted to replace the standard convolution in YOLOv11n (Zhang et al., 2023). AKConv dynamically adjusts kernel sampling positions, reducing parameters while improving feature alignment with target shapes. Given that the coffee bean maturity detection task needs to be carried out outdoors in real time, and to further meet the requirements of detection accuracy and lightweight model deployment.

**Figure 3:**
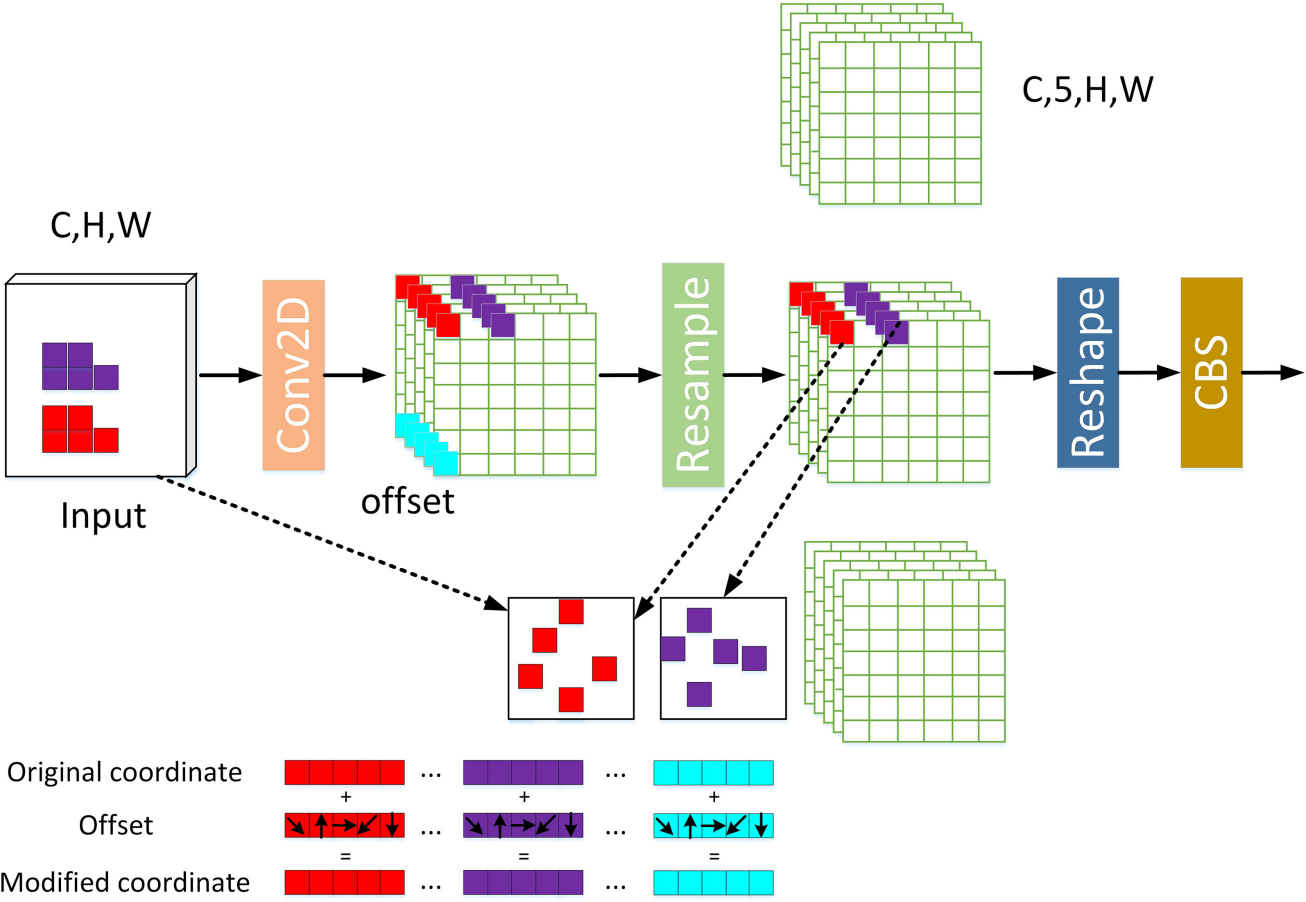
The structure of AKConvolution.

The standard convolution operation is restricted within a local window and cannot capture information from other positions, and its sampling shape is fixed. Furthermore, the size of the convolution kernel is fixed as 3×3 or 5×5, and the number of parameters grows quadratically with the increase in kernel size. The AKConv allows the kernel to have an arbitrary number of parameters and sampling shapes. Details are shown in **Figure 3**.

As shown in **Figure 3**, the original coordinates of the feature map are dynamically adjusted for sampling positions through Offset Learning after 2D convolution. Offset Learning employs an auxiliary convolutional layer to predict the offset for each sampling point, and adds the offset to the initial coordinates to obtain the adjusted sampling position. Then, bilinear interpolation is used to obtain the modified coordinates. To adapt to irregular sampling shapes, the feature map of AKConv is expanded in the spatial dimension, and row/column convolutions are used to extract features, as shown by the red, purple, and blue feature vectors in the figure. Finally, 3D convolution (kernel size of N×1×1) is used for dimensionality reduction to ensure that the input and output feature maps have the same shape.

**Figure 4:**
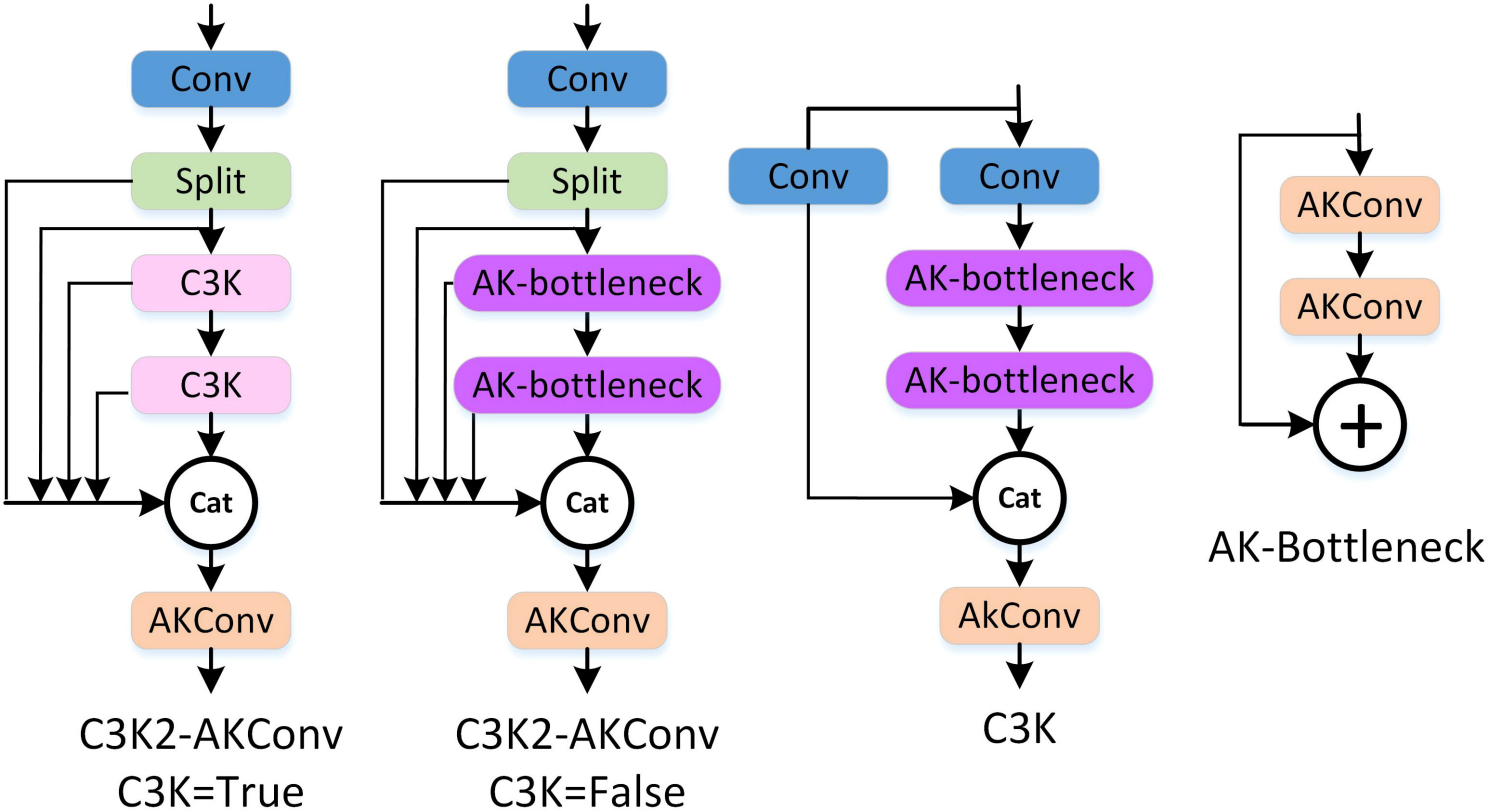
The structure of AKC3K2T/F.

In the YOLOv11 architecture, in addition to the standard convolution modules, its unique C3K2 module also contains a large number of convolutions. Therefore, in addition to replacing the standard convolution modules, the C3K2 module is further replaced to improve the lightweight performance of the model. The structures of AKC3K2 are shown in **Figure 4**.

The C3K2 module in YOLOv11n balances efficiency and performance via partial feature bypass and deep processing. We replace its standard bottleneck convolutions with Arbitrary Kernel Convolution (AKConv), forming an AK-bottleneck with two AKConv layers that adaptively adjust sampling locations via offset learning—enhancing robustness to irregular shapes and occlusions. The final 1×1 convolution in AKConv is adjusted to match internal channel dimensions, and the original output integration layer (fusing shortcut and bottleneck features) is also replaced with AKConv, yielding the AKC3K2 module. This reduces parameters while strengthening local feature extraction through adaptive kernels. Two variants are defined: AKC3K2T (C3K=True) with two AK-bottlenecks, and AKC3K2F (C3K=False) without them—both preserving YOLOv11n’s efficiency–performance trade-off while improving robustness and enabling lightweighting for coffee fruit detection.

To sum up, to adapt to different changes of targets, AKConv adjusts the sampling positions of irregular convolution kernels through the obtained offsets, thus improving the accuracy of feature extraction and the detection precision. Meanwhile, AKConv supports linearly increasing or decreasing the number of convolution parameters, which is helpful for optimizing performance in hardware environments and is especially suitable for lightweight applications of coffee fruit detection.

### 2.4 The structure of single scale ViT

Indeed, the Transformer model relies on a global attention mechanism that requires substantial computational resources for optimal performance (Vaswani et al., 2017). Consequently, it becomes crucial to address this issue effectively. To mitigate this concern, the initial image or multi-layer feature maps are eschewed as input, and instead only the final feature map obtained from the backbone is used. This is then directly connected to the neck. Additionally, we retain only two key components of the ViT: the Position Embedding and the Encoder, both of which are improved (Han et al., 2022).

To enhance the detection accuracy, an enhanced global attention mechanism based on the Vision Transformer model is introduced. This modification takes into consideration that some coffee fruit maturity may share similarities, while others have limited occurrence areas. By incorporating this improved global attention mechanism, the detection accuracy can be further improved in detecting different maturity.

**Figure 5:**
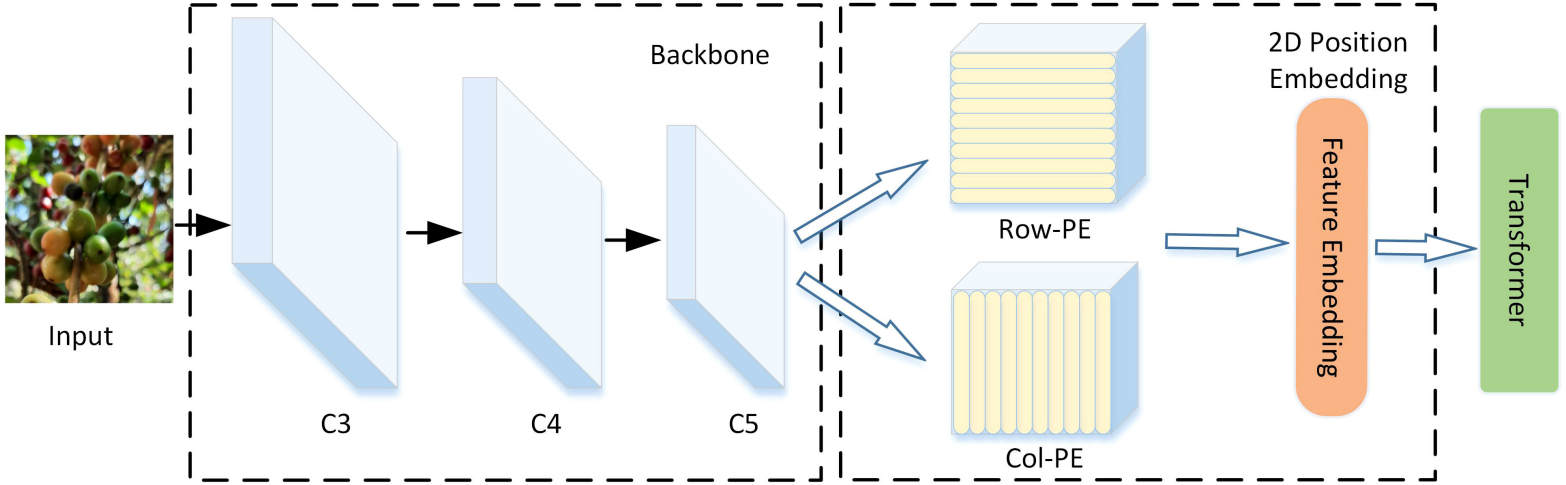
The structure of 2D position embedding (2D PE).

The current common DETR algorithms extract the last three layers of feature maps (C3, C4, C5 in **Figure 5**) from the backbone network as the input for ViT. However, this approach usually has two problems:

1) Previous DETRs, such as Deformable DETR (Zhu et al., 2020), flatten multi-scale features and concatenate them into a single long sequence vector. This approach enables effective interaction between the different scale features, but it also introduces significant computational complexity and increases the time required for processing.

2) Compared to the shallower C3 and C4 features, the deepest layer C5 feature has deeper, higher-level, and richer semantic features. These semantic features are more useful for distinguishing different objects and are more desirable for Transformer. Shallow features do not play much of a role due to the lack of better semantic features.

To address these issues, only the C5 feature map output by the backbone is used as the input for the ViT. To retain key feature information, we replace feature map flattening into a vector with 2D encoding in the Position Embedding (2D PE) module. Additionally, a lightweight single-scale ViT Encoder (SSVE) is adopted, called Single Scale ViT (SSViT).

The MHSA (Multi-Head Self-Attention) aggregation in Transformer combines input elements without differentiating their positions; thus, Transformer possess permutation invariance. To alleviate this issue, we need to embed spatial information into the feature map, which requires adding 2D PE to the final layer feature map. Specifically, the original sine and cosine positional encodings in Position Embedding are respectively extended to column and row positional encodings, and concatenated with them finally. Details are shown in **Figure 5**.

**Figure 6:**
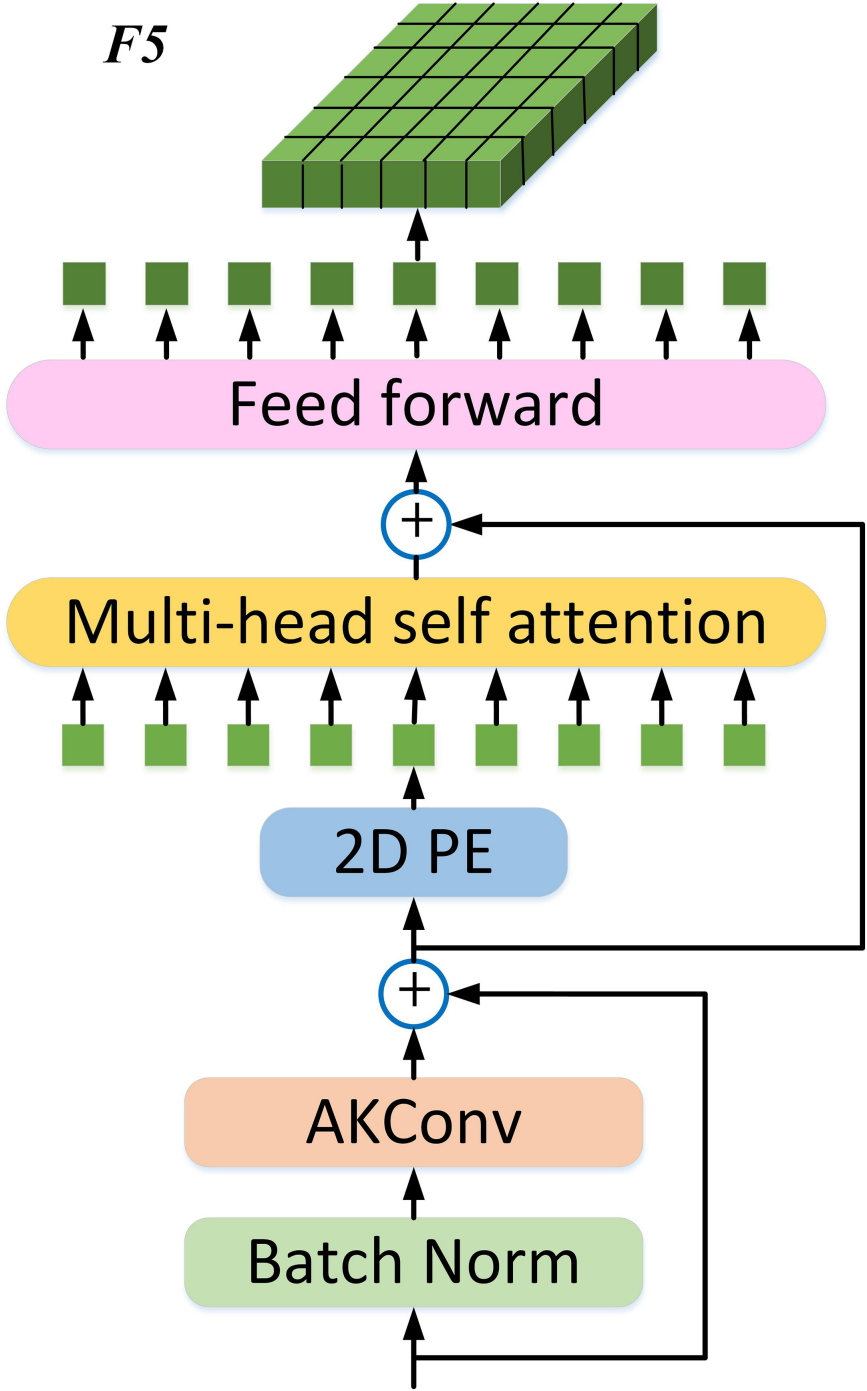
The structure of Single Scale ViT (SSViT).

The final feature map C5 obtained from the backbone first connects to BN (batch norm) and AKConv, and then forms a residual connection with C5, further enhancing feature robustness and information integrity. To further improve the real-time performance of the Transformer, after the feature map is processed with 2D PE, a single-scale ViT Encoder (SSVE) is used, which only contains one Encoder layer (Multi-Head Self-Attention + Feed Forward network) to process the output of Q, K, and V at three scales. Note that the three scales share one SSVE, and through this shared operation, the information of the three scales can interact to some extent. This output feature and the input of the 2D PE module complete the residual connection again. Finally, the processing results are concatenated to form a vector, which is then adjusted back to a 2D feature map, denoted as F5. In the neck, C3, C4, and F5 are sent to neck for multi-scale feature fusion. See **Figure 6** for details.

### 2.5 The structure of MSCA-head

In coffee fruit maturity detection, target fruits often exhibit significant scale variation: they range from densely clustered small fruits to relatively isolated larger ones, frequently suffering from occlusion by leaves or overlapping with neighboring fruits, which leads to ambiguous local features. Traditional detection heads struggle to retain small object local features, while large kernels lose local information—MSCA uses 5×5 small kernels to capture these details and large strip convolutions for global context. Motivated by the advantages of multi-scale feature representation, we integrate a Multi-scale Convolutional Attention (MSCA) module into the three detection heads of different scales (large, medium, and small) in YOLOv11 (Guo et al., 2022). Convolutional kernels of different sizes can capture spatial context at varying ranges. Small kernels focus on local details, aiding precise localization of small targets, while larger kernels expand the receptive field, enhancing robustness against occlusion and deformation.

**Figure 7:**
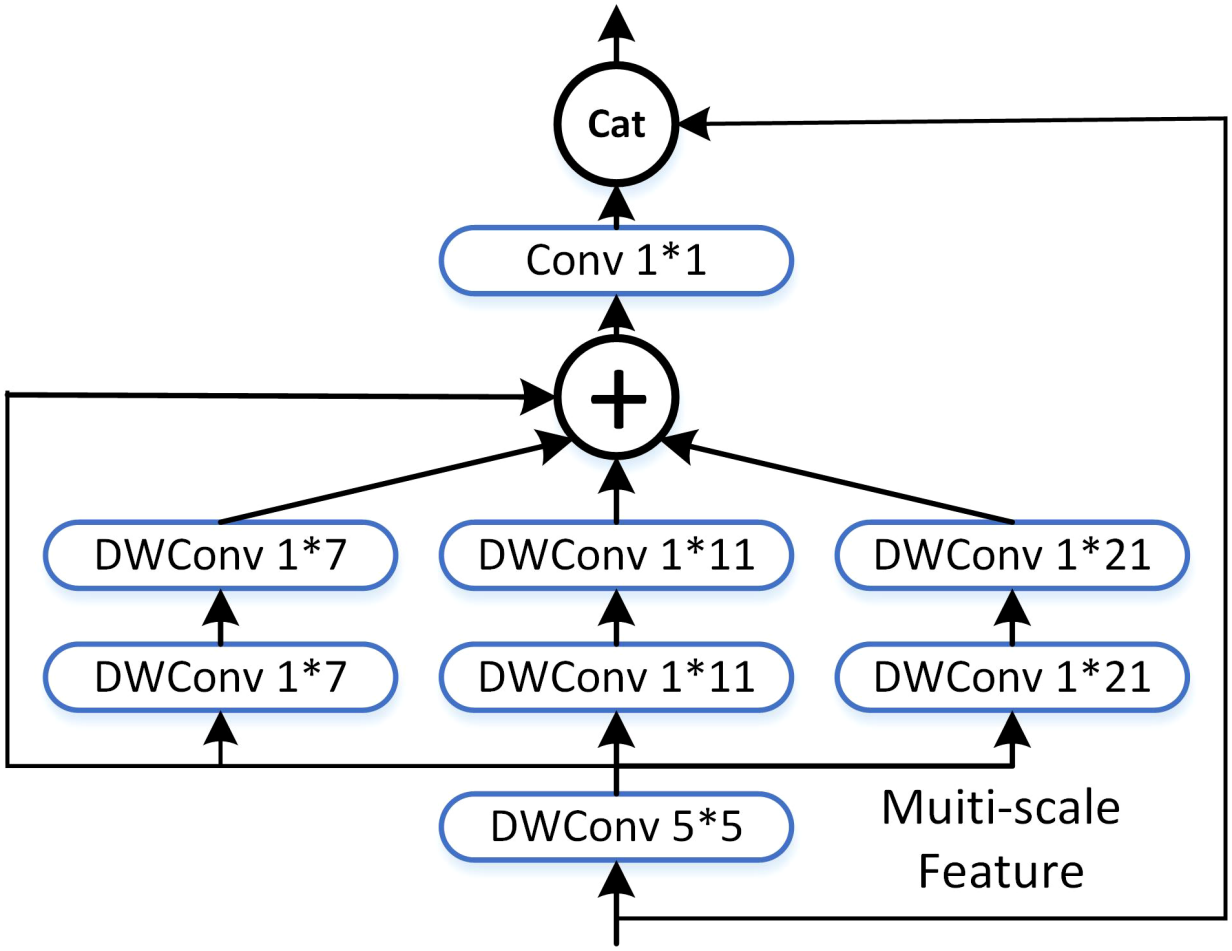
The structure of MSCA.

By deeply integrating MSCA with the detection heads, each scale-specific branch gains the ability to adaptively attend to critical regions, improving detection accuracy of multi-scale and occluded coffee fruits without substantially increasing computational cost. Therefore, the MSCA-Head is designed to enhance the model’s perceptual ability in complex agricultural scenarios through a lightweight, multi-scale attention mechanism. See **Figure 7** for details.

MSCA consists of three parts: depth-wise convolution to aggregate local information, multi-branch depth-wise strip convolution to capture multi-scale contexts, and 1×1 convolution is used for dimension reduction and feature fusion. The details of MSCA are shown in Equations 1 and 2:

(1) $$Att={\text{Conv}}_{1\times 1}(\sum_{i=0}^{3}{\text{Scale}}_{i}(\text{DW}-\text{Conv}(F)))$$

(2) Out=Att⊗F

In Equations 1 and 2, *F* represents the input feature. DW-Conv denotes depth-wise separable convolution. ${\text{Scale}}_{i}$ represents a total of 4 branches with different scales, and the kernel sizes of each branch are set to 5, 7, 11, and 21 respectively. In 3 of these branches, two depth-wise strip convolutions are used to simulate large kernels to further reduce the amount of computation, facilitating outdoor real-time detection. For example, a pair of 7×1 and 1×7 convolutions are used to simulate a 7×7 convolution as shown in **Figure 7**. ${\text{Conv}}_{1\times 1}$ denotes a 1×1 convolution applied after multi-scale feature fusion to reduce dimensionality, resulting in an output feature dimension that matches the input feature dimension. ⊗ is the element-wise matrix multiplication operation. *Att* and *Out* are the attention map and output respectively.

**Figure 8:**
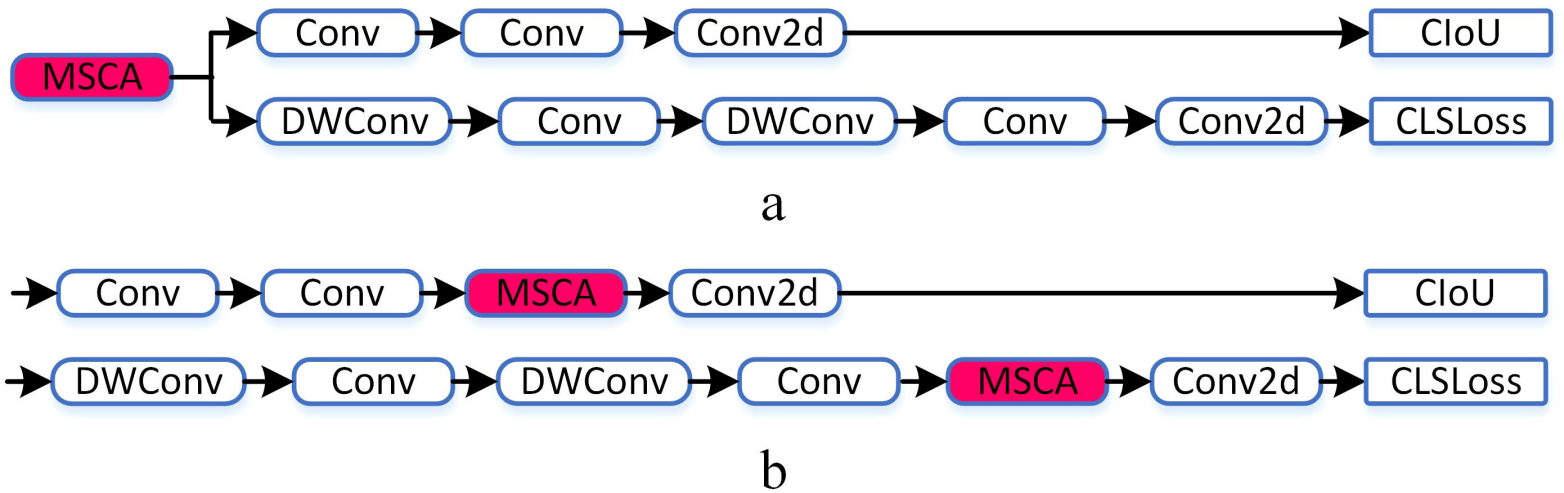
Two different MSCA-head.

This paper mainly designs two ways of fusing MSCA with the head, as detailed in **Figure 8**. In **Figure 8a**, MSCA is added after the three feature maps of different scales extracted by the neck and before the head branch; In **Figure 8b**, MSCA is added after the standard Conv of the two branches. For specific effects, refer to the comparative experiment section below.

In summary, the extraction of multi-scale convolutional features by MSCA enables the model to better handle objects of different scales. Moreover, depth-wise separable convolutions are used and combined with strip convolutions to approximate large-kernel convolutions, ensuring the model is lightweight and improving the detection accuracy.

### 2.6 Experimental environment

The experimental hardware environment is configured with Intel I7–13700 CPU; 32GB RAM; GEFORCE RTX3070 GPU. The operating system is Windows10 professional edition, the programming language is Python 3.8, and the acceleration environment is CUDA 11.1, CUDNN 8.2.0. The training parameters are shown in **Table 2**.

**Table 2:** The implementation details of training parameters.

Parameter	Value	Parameter	Value
optimizer	AdamW	Weight Decay	0.0005
Learning Rate	0.001	Momentum	0.937
Batch Size	8	warmup steps	300
Image Size	640×640	Epochs	200

### 2.7 Evaluation metrics

Model performance is evaluated using a combination of detection metrics. Equations 3–5 are the calculation formulas for *Precision* (P), *Recall* (R), and mean Average Precision (*mAP*), which mainly reflect the detection performance of the model. Parameters and Frames per second (FPS) mainly reflect the lightweight and real-time performance of the model. These are the most direct evaluation metrics for achieving real-time outdoor detection of coffee fruit ripeness.

(3) $$Precision=\frac{TP}{TP+FP}$$

(4) $$Recall=\frac{TP}{TP+FN}$$

(5) $$mAP=\frac{1}{T}\sum_{t=1}^{T}AP_{t}$$

Where *Precision* is defined as the ratio of True Positives (*TP*, the number of correctly predicted positive instances) to the sum of True Positives and False Positives (*FP*, the number of incorrectly predicted positive instances from negative ones), measuring the accuracy of positive predictions in detection. *Recall* represents the ratio of *TP* to the sum of *TP* and False Negatives (*FN*, the number of incorrectly predicted negative instances from positive ones). It quantifies how well a model can identify actual positive instances. *mAP* measures a model’s overall performance in multi-category detection. *T* is the total number of categories, *T=5* is the total number of classes (dry, overripe, ripe, semi_ripe, unripe). $AP_{t}$ is the Average Precision for the *t-th* one.

## 3 Experimental results

### 3.1 Ablation study on AKC3K2

The proposed SSViT-YOLOv11 mainly consists of three modules: AKC3K2, SSViT, and MSCA-head, so the influence of each improved module on the model performance is verified separately.

In this section, the AKConv and AKC3K2 are verified through experiments. The following tests are conducted on the baseline of YOLOv11n: 1. Only replacing Conv with the AKConv module; 2. Only replacing C3K2True with the AKC3K2True module; 3. Only replacing C3K2False with the AKC3K2False module; 4. Replacing both C3K2 (True & False) modules completely; 5. Replacing all convolutions with AKConv. The FPS in the table represents the results of the model deployed on Raspberry Pi 5. Details are shown in **Table 3**.

**Table 3:** The results of the ablation study on AKC3K2.

Methods	AKConv	AKC3K2T	AKC3K2F	mAP@0.5	Params (M)	FPS
1				77.14	2.62	20
2	✓			78.85	2.17	22
3		✓		79.41	1.95	25
4			✓	79.83	2.12	22
5		✓	✓	**80.22**	1.57	26
6	✓	✓	✓	78.50	**1.02**	**27**

Bold font indicates the optimal parameter.

On the basis of YOLOv11n, compared by integrating AKConv/AKC3K2T/AKC3K2F modules increases mAP by 1.71%/2.27%/2.69%, the number of parameters is greatly reduced 17.2%/25.6%/19.1%, the FPS is improved by 2/5/2 frames respectively. These results show each individual module effectively improves detection accuracy, reduces model size, and boosts inference speed. The combination of AKC3K2T and AKC3K2F enables the model to achieve the optimal mAP of 80.22% (+3.08%). Moreover, compared with adding the modules individually, both the Params and FPS are further improved. When all three modules are replaced, it may be because the YOLOv11n is already a very lightweight architecture, and over-optimization leads to a decrease instead of an increase in mAP. Additionally, an FPS of 27, compared with an FPS of 26 for the AKC3K2 combination, only increases by 1 frame, which has a very small impact on real time detection. Considering comprehensively, we selected the AKC3K2 combination structure with higher accuracy.

### 3.2 Ablation study on SSViT

In this section, the 2D PE and SSVE are verified through experiments first. The following tests are conducted on the baseline of YOLOv11n: 1. Only replacing 1D PE with 2D PE; 2. Only replacing multi-scale ViT encoder (MSVE) with the SSVE; 3. Replacing both ViT modules with SSViT completely. Details are shown in **Table 4**.

**Table 4:** The results of the ablation study on 2D PE & SSVE.

Methods	2D PE	SSVE	mAP@0.5	Params (M)	FPS
1			77.14	**2.62**	**20**
2	✓		79.62	3.23	6
3		✓	79.46	2.74	16
4	✓	✓	**79.89**	2.74	16

Bold font indicates the optimal parameter.

As can be seen in **Table 4**, on the basis of YOLOv11n, compared by integrating the 2D PE + MSVE module, the accuracy has improved by 2.48%, but the number of parameters has increased by 23.3%, and the FPS has dropped significantly by 70.0%. This is mainly because the traditional ViT includes 12 encoders, which leads to a substantial increase in both parameter count and computational complexity. Although it improves detection accuracy, it has a significant negative impact on real-time outdoor detection. The 3rd and 4th groups of ablation experiments added the 1D PE + SSVE module and 2D PE + SSVE module respectively. The accuracy rates of the two groups of experiments increased by 2.32% and 2.75% respectively, with the parameter counts only increasing by 4.6% and the FPS slightly decreasing by 20.0% in both cases. Explanations: 1. Compared with 1D PE, 2D PE achieves higher detection accuracy, and has almost no impact on both the model parameter count and FPS. 2. The SSVE module achieves a comparable improvement in detection accuracy to the MSVE module, but shows significant improvements in terms of parameter count and FPS. Considering detection accuracy, lightweight performance and real-time capability, 2D PE + SSVE is the optimal improvement scheme.

The structure of the SSViT module shown in **Figure 6**, in addition to the core 2D PE & SSVE, this paper also incorporates AKConv and residual connections. Through ablation experiments, the following tests were conducted on the baseline of YOLOv11n + 2D PE + SSVE in **Table 4**: 1. Only adding Conv in the SSViT; 2. Only adding AKConv in the SSViT; 3. Adding Conv with residual connections; 4. Adding AKConv with residual connections. Details are shown in **Table 5**.

**Table 5:** The results of the ablation study on AKConv & residual connections.

Methods	Conv	AKConv	residual	mAP@0.5	Params (M)	FPS
1				79.89	**2.74**	16
2	✓			79.99	2.90	15
3		✓		80.12	2.82	16
4	✓		✓	80.04	2.90	15
5		✓	✓	**80.36**	2.82	**16**

Bold font indicates the optimal parameter.

As can be seen from **Table 5**, after adding the Conv/AKConv modules, the detection accuracy increased by 0.1%/0.23%, the number of parameters increased by approximately 6%/3%, and the FPS remained almost unchanged. When both modules were combined with the residual connection structure, the detection accuracy increased by 0.15%/0.47%, with no changes in the number of parameters and FPS. This indicates that the addition of AKConv and residual connections to SSViT is more conducive to improving feature extraction capability, resulting in the optimal mAP value. Moreover, the increase in model parameters is minimal and there is no change in FPS.

### 3.3 Ablation study on the MSCA-head

In this section, the MSCA is verified through experiment. The following tests are conducted on the baseline of YOLOv11n: 1. Adding MSCA in front of the head (**Figure 8a**); 2. By adding MSCA in middle of the head (**Figure 8b**). Details are shown in **Table 6**.

**Table 6:** The results of the ablation study on the MSCA-Head.

Methods	MSCA-a	MSCA-b	mAP@0.5	Params (M)	FPS
1			77.14	**2.62**	**20**
2	✓		**79.12**	2.93	19
3		✓	77.85	3.20	17

Bold font indicates the optimal parameter.

As can be seen from **Table 6**, after adding the MSCA-a/MSCA-b module, the detection accuracy increased by 1.98%/0.71%, the number of parameters increased by approximately 11.8%/22.1%, and the FPS decreased by 1/3 frames respectively.

It is demonstrated that MSCA effectively improves the detection accuracy of objects at different scales. Meanwhile, due to its approximate large-kernel convolutions, the model does not increase much computational complexity, ensuring the real-time performance of detection. MSCA, when added before the head, not only achieves higher accuracy but also performs better in terms of parameter count and FPS metrics. Therefore, the structure shown in **Figure 8a** is finally adopted in this paper.

### 3.4 Ablation study on the overall improvement

In this section, the three core modules AKC3K2, SSViT, MSCA-head are verified through experiments. Since the ablation experiments conducted earlier have thoroughly verified the performance of each module, this section will mainly focus on validating the network performance of different module combinations. The following tests are conducted on the baseline of YOLOv11n. Details are shown in **Table 7**.

**Table 7:** The ablation study on overall improvement.

Method	AKC3K2	SSViT	MSCA	P	R	mAP@0.5	Params	FPS
1				0.729	0.681	77.14	2.62	20
2	✓			0.733	0.701	80.22	**1.57**	**26**
3		✓		0.762	0.755	80.36	2.82	16
4			✓	0.743	0.699	79.12	2.93	19
5	✓	✓		0.788	0.750	83.31	1.77	23
6	✓		✓	0.753	0.756	80.42	1.82	25
7		✓	✓	0.776	0.761	82.45	3.19	15
8	✓	✓	✓	**0.811**	**0.774**	**84.54**	2.16	23

Bold font indicates the optimal parameter.

As can be seen in **Table 7**, on the basis of YOLOv11n, compared by integrating the AKC3K2/SSViT/MSCA, the detection accuracy increased by 3.08%/3.22%/1.98%, the changes in the number of parameters are -40.1%/+7.6%/+11.8%, and the changes in FPS are +6/-4/-1 frames respectively. This indicates that Transformer achieves the best improvement in model performance. AKC3K2 not only enhances performance but also significantly realizes model lightweighting. MSCA results in the largest increase in the number of model parameters, yet the FPS is not significantly affected. Among the combination effects of different modules, the AKC3K2+MSCA combination shows the smallest improvement in precision.

Among all module combinations, AKC3K2+MSCA yields the smallest precision improvement, while SSViT+MSCA leads to the most parameter-heavy model (3.19M) and the lowest FPS (15). In contrast, the full integration of all three modules (Method 8) achieves the optimal balance, attaining peak values in precision (0.811), recall (0.774), and mAP@0.5 (84.54%), while simultaneously reducing parameters by 17.6% (from 2.62M to 2.16M) and increasing FPS by 3 compared to the baseline. This confirms that our proposed architecture not only enhances detection capability but also maintains efficiency suitable for real-world deployment.

### 3.5 Comparison with other detectors

**Table 8** compares SSViT-YOLOv11 with other YOLO detectors: YOLOv7t (Wang et al., 2023), YOLOv8n (Sohan et al., 2024), YOLOv9t (Wang et al., 2024b), YOLOv10n (Wang et al., 2024a), YOLOv11n, and ViT detectors: Swin-Transformer (Liu et al., 2021), YOLOv8n-rtdetr (Zhao et al., 2024)), also selected two high performing baseline models (Eron et al., 2024; Kazama et al., 2024) from **Table 9** for comparison.

**Table 8:** The comparison results of different methods.

Method	P	R	mAP@0.5	Params	FPS
YOLOv7t	0.693	0.701	74.42	6.02	9
YOLOv8n	0.743	0.692	76.03	3.15	12
YOLOv9t	0.719	0.697	74.50	**2.01**	14
YOLOv10n	0.735	0.730	78.87	2.83	18
YOLOv11n	0.729	0.681	77.14	2.62	20
Swin-Transformer	0.788	0.721	81.57	7.23	5
YOLOv8n-rtdetr	0.794	0.756	82.68	3.16	11
Eron et al. (2024)	0.803	0.745	82.12	36.9	1
Kazama et al. (2024)	0.747	0.714	78.01	3.19	12
SSViT-YOLOv11	**0.811**	**0.774**	**84.54**	2.16	**23**

Bold font indicates the optimal parameter.

**Table 9:** Comparative analysis of related coffee fruit maturity detection studies.

Author, year, model	Key performance metrics	Core advantages	Main limitations	Advantages of SSViT-YOLOv11n
Bazame et al.(2023), YOLOv4	At a resolution of 320×320, the mAP of YOLOv4t/3t: 68%/40%; At a resolution of 800×800, the mAP of YOLOv4t/3t: 79%/77%.	Performs better in detecting green unripe fruits at high resolutions.	1. Limited to 3 classes: overripe, ripe, and unripe; 2. Poor small-object performance: the highest mAP achieved remains below 80%; 3. >12% mAP drop in shade.	1. Achieves an overall mAP of 84.54% across 5 maturity categories 2. The MSCA module enhances multi-scale feature fusion, improving the model’s capability to detect small targets; 3. SSViT + AKConv enhance lighting robustness (only ~3.7% drop in dim light);
Eron et al. (2024), YOLOv7	Detects five classes (unripe, yellow, cherry, raisin, and dry), with mAP: 0.605, Precision: 0.627, Recall: 0.682, Parameters: 36.9M.	Refined the model’s detection categories.	1. Very large model (36.9M params), difficult to deploy on mobile devices; 2. Recall fluctuating by ±7% under severe occlusion; 3. The detection performance (mAP) is relatively poor.	1. Lightweight Model Design: With only 2.16M parameters, and specifically optimized for edge devices; 2. MSCA + SSViT achieve an mAP of 75.8% under severe occlusion and control recall fluctuation within ±3%; 3. Balanced Accuracy and Efficiency: mAP increased to 84.54% while maintaining the speed of 23 FPS.
Ulhaq et al. (2024), Multispectral + Bayesian Method	Classification accuracy: 91.65%, Recall: 92.05%, F1-score: 90.93%; Inference speed: 1100 FPS	Extremely fast inference speed, runs efficiently on CPU, and is highly suitable for embedded deployment.	1. Relies on multispectral data, requiring specialized and costly hardware; 2. Supports image classification tasks only. 3. High false positive rate (increased by 38%) in complex outdoor backgrounds	1. RGB-only solution: Eliminates dependency on multispectral hardware, enhancing accessibility and general applicability; 2. More challenging task: Maturity detection based on YOLOv11, which is more complex in applications. 3. AKConv dynamically avoids background interference, reducing the false positive rate by more than 25% in complex backgrounds.
Kazama et al.(2024), Yolov8n	mAP@0.50 of 74.20%, Parameters: 3.19M	Images were captured under varying lighting and canopy conditions.	1. Fewer maturity classes (unripe, semi-ripe, ripe, overripe); 2. The detection accuracy is relatively low; 3. No explicit occlusion/lighting robustness design	1. Support detection across five distinct maturity; 2. Comprehensive performance improvement: Our proposed algorithm achieves higher mAP (84.54%); 3. Integrates AKConv, SSViT, and MSCA, with mAP dropping by only 8.5% under strong light and performance decline controlled within 13.8% under severe occlusion.
Tamayo-Monsalve et al.(2022), Inception-ResNet + multispectral	Classification accuracy > 98%, Processing time > 200 ms per image, multispectral camera (costing over $15,000), controlled lighting environment	Achieves extremely high classification accuracy by leveraging spectral information.	1. Extremely high hardware cost; 2. Not real-time (<5 FPS); 3. Accuracy drops by more than 20% when leaving the controlled lighting environment.	1. Low cost: Requires RGB camera only, lowering the deployment barrier. 2. Real-time performance: Achieves an inference speed of 23 FPS in real outdoor scenes. 3. Strong robustness: maintains high accuracy under complex lighting conditions such as strong light, shadows, and cloudy days.

Compared to YOLO detectors YOLOv7t/YOLOv8n/YOLOv9t/YOLOv10n/YOLOv11n, our method significantly improves mAP by 10.12%/8.51%/10.04%/5.67%/7.40%, increases FPS by 14/11/9/5/3, and the changes in the number of parameters are –64.1%/–31.4%/+7.4%/–23.7%/–17.6%. Except for being slightly inferior to YOLOv9t in terms of the number of parameters, it achieves the best performance in all other metrics. Compared to ViT detectors Swin-Transformer/YOLOv8n-rtdetr, our method improves accuracy by 2.97%/1.86% mAP, increases FPS by 18/12 frames, and the number of parameters decreased by 70.1%/31.6%. Furthermore, SSViT-YOLOv11 outperforms Eron et al. (2024) and Kazama et al. (2024) in mAP@0.5 by 2.42% and 6.53%, respectively, while using 94.2% and 32.3% fewer parameters and achieving 23× and 1.9× higher FPS. These results demonstrate that SSViT-YOLOv11 surpasses both YOLO and real-time Transformer-based detectors in accuracy, speed, and model efficiency.

To evaluate the detection accuracy of different algorithms under three distinct real-world conditions, specifically: **Lighting intensity:** 1. Normal: Fruits are clearly visible; 2. Bright: Direct sunlight shines on the fruit surface, causing bright specular highlights or glare; 3. Dim: Images captured under leaf shade or cloudy conditions. Occlusion level: 1. No occlusion: The fruit is fully visible; 2. Light occlusion: Less than 30% of the fruit area is occluded (e.g., by leaves); 3. Severe occlusion: The coffee fruit is covered by multiple leaves or heavily overlapped by neighboring fruit cluster, with less than 50% of its surface visible. Object scale variation: Based on bounding box height/width in pixels at 640×640 input resolution. 1. Small fruit: < 20 pixels; 2. Medium fruit: 20–40 pixels; 3. Large fruit: > 40 pixels. Furthermore, representative detection algorithms listed in **Table 8** are included in the comparison for comprehensive analysis. For each category, 50 images are selected. Detection performance under each condition is reported in **Table 10**.

**Table 10:** The comprehensive performance (mAP@0.5) across different conditions.

Method	Lighting(mAP@0.5)			Occlusion(mAP@0.5)			Scale(mAP@0.5)		
	Normal	Dim	Bright	No	Light	Severe	Small	Medium	Large
YOLOv10n	82.1	77.3	70.6	84.5	75.2	61.8	60.9	79.4	86.3
YOLOv11n	83.7	79	72.4	86.2	78.5	65.3	61.8	82.4	89.1
Swin-Transformer	85.3	80.1	73.2	87	79.6	64.1	66.2	83	88.7
YOLOv8n-RTDETR	84.9	79.8	71.9	86.8	77.9	67.5	68.5	81.7	88.9
SSViT-YOLOv11 (Ours)	86.4	82.7	77.9	89.6	84.3	75.8	71.5	86.2	91.4

As shown in **Table 10**, regarding Lighting robustness: All methods exhibit the most significant performance degradation under Bright conditions, as highlight regions cause color distortion that interferes with maturity assessment. SSViT-YOLOv11 achieves the best results across all three lighting conditions, with only a 3.7% drop in mAP@0.5 from Normal to Dim, substantially outperforming other methods (which average a ~5% decline). Occlusion Robustness: Severe occlusion presents the greatest challenge, causing a performance drop of over 19% for all baselines. SSViT-YOLOv11 attains 75.8% mAP@0.5 under severe occlusion, surpassing the second-best method (YOLOv8n-RTDETR) by 8.3 percentage points. Moreover, it shows the smallest performance drop (only 13.8% from no occlusion to severe occlusion), demonstrating exceptional robustness to occlusion. Scale Sensitivity: Detecting small fruits remains a common bottleneck, with all methods achieving their lowest scores in this category. SSViT-YOLOv11 reaches 71.5% mAP@0.5 on small fruits, outperforming the next-best method (RTDETR) by 3.0% and the YOLOv11n by 9.7%. It also maintains leading performance on medium and large fruits. Technical attribution: 1. AKC3K2: Dynamically adjusts convolution sampling points to bypass occluders and highlight regions. 2. SSViT: Leverages global attention to infer the presence and location of occluded fruits and compensates for local illumination distortions. 3. MSCA-Head: Employs multi-scale Conv. to effectively enhance responses for small objects and reduce false detections.

### 3.6 Performance visualization of YOLOv11n & SSViT-YOLOv11

**Figure 9:**
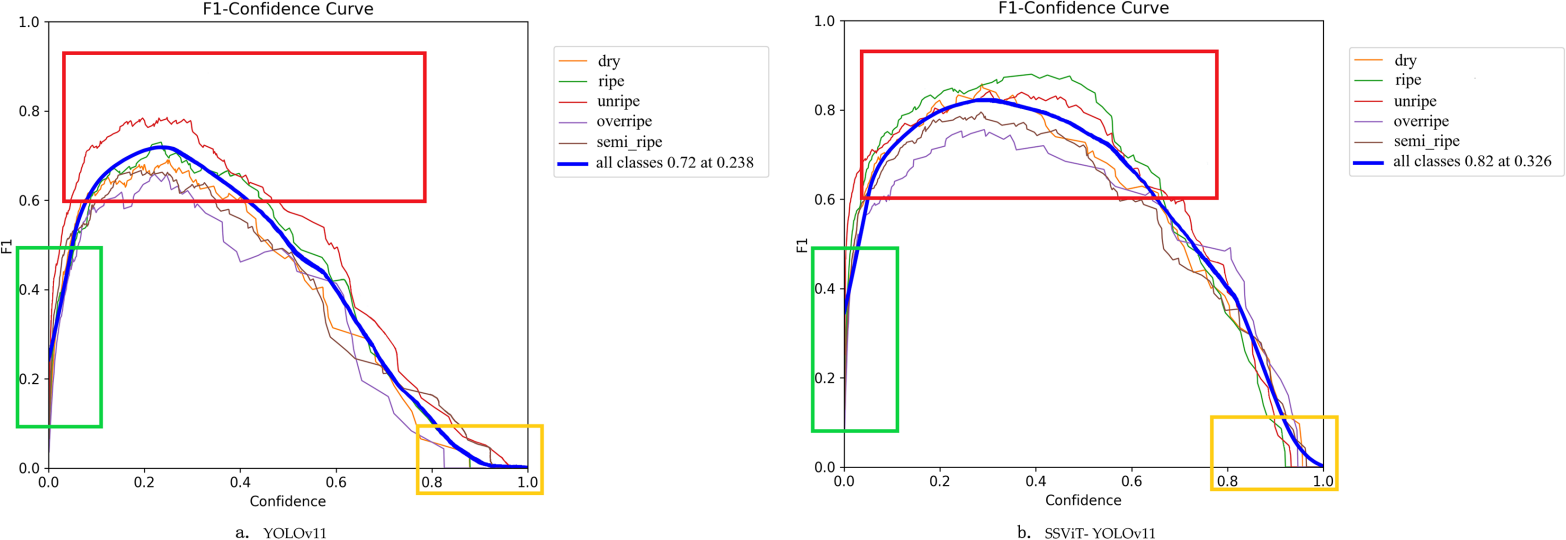
The F1-confidence curves of YOLOv11n **(a)** and SSViT- YOLOv11 **(b)**.

**Figure 9** presents the F1-confidence curves of YOLOv11n and SSViT-YOLOv11. In the figure, three rectangular boxes of different colors are used to mark the regions with obvious contrast.

In the green box region (confidence threshold below 0.1), the F1 score of SSViT-YOLOv11 is consistently higher than that of YOLOv11n. This indicates that the model proposed in this paper can more effectively detect small-scale or occluded fruit targets while retaining low-confidence predictions. In the red box region (confidence threshold approximately 0.2–0.8), the F1 score of SSViT-YOLOv11 shows a significant improvement for all categories except for the unripe category, where the F1 score of targets with distinct features remains relatively unchanged. In the yellow box region (confidence threshold > 0.8), the F1 score of the proposed method decreases more slowly. This demonstrates that the model has stronger robustness and the problem of overconfidence in misclassifications is alleviated.

**Figure 10:**
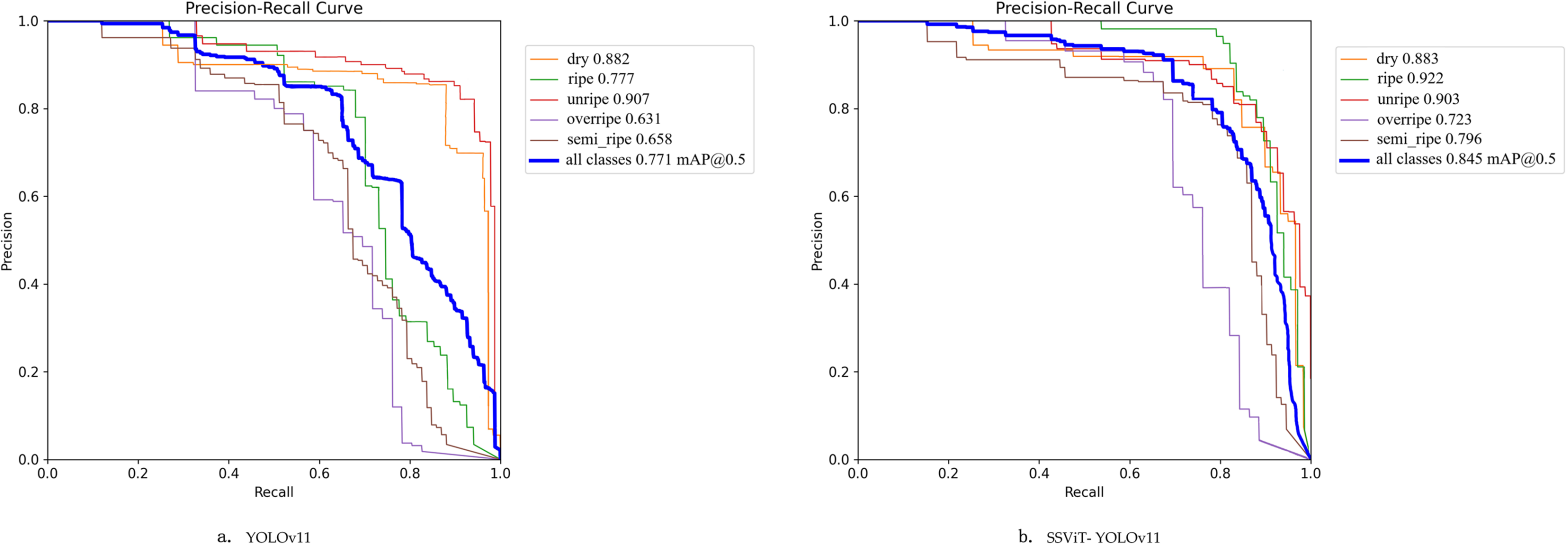
The P-R curves of YOLOv11n **(a)** and SSViT- YOLOv11 **(b)**.

**Figure 10** presents the Precision–Recall (PR) curves of YOLOv11n and SSViT- YOLOv11. Compared with the YOLOv11n and SSViT-YOLOv11, the AP values for the dry category are 0.882 and 0.883, and for the unripe category are 0.907 and 0.903, which are extremely close. This indicates that there is little difference between the two algorithms in capturing and distinguishing the salient features of these two categories. For the ripe category, the AP values are 0.777 and 0.922, with the most significant increase. The AP values for the overripe and semi-ripe categories also show obvious improvements. This shows that the SSViT-YOLOv11 optimizes the feature extraction and classification mechanism through improved modules, and can more accurately mine the differential features between them and fruits of other maturity stages. It effectively avoids confusion with other categories due to appearance, shape, color and other features. The mAP@0.5 of SSViT-YOLOv11 reaches 0.845, showing a substantial improvement compared to YOLOv11n. From the curve, as the recall rate changes from low to high, the precision decreases more gently. These indicate that SSViT-YOLOv11 model performs better in balancing the precision and comprehensiveness of detecting fruits at different maturity stages, and has superior overall detection performance.

Overall, the performance improvement of SSViT-YOLOv11 is closely related to its architectural enhancements. By replacing the AKC3K2 module, it can better extract features of fruits in different states, sizes and shapes through deformable convolutions. Meanwhile, the incorporation of the SSViT module enables the model to better capture global features, which is particularly important for processing large areas of clustered fruit regions. Additionally, the introduction of the MSCA module in the head part, which combines attention mechanisms from convolutions of different scales, allows for better detection of coffee fruits of varying sizes and those that are occluded. These improvements, when compared with YOLOv11, demonstrate that SSViT-YOLOv11 has stronger accuracy and robustness in practical deployment.

**Figure 11:**
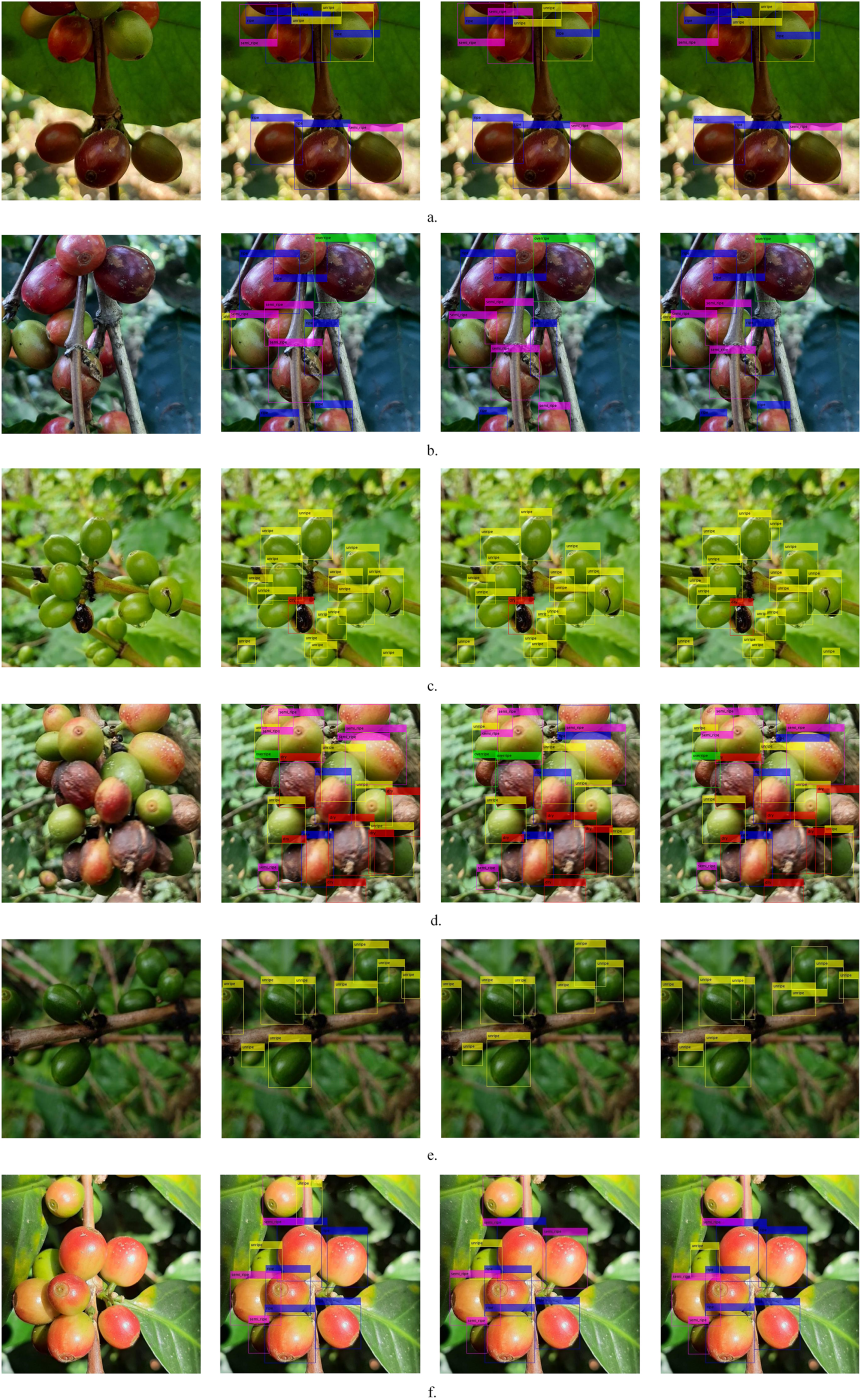
The detection results of YOLOv11n & SSViT-YOLOv11. Lighting: Groups **(a)** and **(e)** feature dim lighting; **(b–d)** have normal lighting; **(f)** exhibits bright lighting. Occlusion: Groups **(b)**, **(c)**, and **(d)** involve heavy occlusion, **(a)**, **(e)**, and **(f)** exhibit light occlusion. Scale: Groups **(a)** and **(b)** contain medium fruits; **(c)** and **(e)** contain small fruits; **(f)** contains large fruits; **(d)** includes a mix of scales. In group **(a)** and **(f)**, YOLOv11n misclassified the ripe fruit. Both of them struggled with highly occluded fruits in dim and bright lighting. In group **(b)** and **(d)**, YOLOv11n produced more misclassifications and missed the heavily occluded fruits. In group **(c)**, it missed a small unripe fruit, ours detected all annotated fruits and even identified an unannotated one. In group **(e)**, both models performed well under dim lighting, though YOLOv11n missed a small fruit.

**Figure 11** shows the actual detection effects of YOLOv11n and SSViT-YOLOv11. Combining the three different outdoor conditions listed in **Table 10**—varying lighting, occlusion, and scale—we selected 6 representative detection comparison image pairs for demonstration. Each group (**a-f**) in **Figure 11** shows the original image, ground truth, the prediction results of the YOLOv11n and SSViT-YOLOv11 respectively. Moreover, these six image groups collectively cover all five fruit maturity stages: dry, overripe, ripe, semi-ripe, and unripe. The annotation and comparative analysis are shown in figure caption. Overall, SSViT-YOLOv11 demonstrates superior robustness, with fewer missed and false detections across diverse lighting, occlusion, and scale conditions.

## 4 Discussion

The proposed SSViT-YOLOv11 demonstrates a favorable trade−off between detection accuracy, inference speed, and model compactness for coffee fruit maturity assessment. On the compiled dataset, the complete model attains precision of 0.811, recall of 0.774, mAP@0.5 of 84.54%, inference speed of 23 FPS and model size of 2.16M parameters.

Synergy analyze: Our ablation studies (**Table 3**) reveal a synergistic effect: while each module alone improves mAP@0.5 by 1.98% (MSCA), 3.08% (AKC3K2), or 2.15% (SSViT), their combination yields a cumulative gain of 5.06%—exceeding the sum of individual contributions. This suggests that SSViT’s global context resolves ambiguities that AKConv cannot (e.g., heavily occluded clusters), while AKConv’s adaptive sampling provides precise local cues that SSViT alone may miss due to its single-scale input. Meanwhile, MSCA amplifies discriminative regions identified by both, particularly for small fruits blending into foliage. However, this integration entails trade-offs. The added modules increase architectural complexity and may introduce hardware-specific latency (e.g., AKConv’s dynamic sampling). Yet, by confining SSViT to C5 and using weight-sharing across scales, we limit parameter growth to +0.54M (vs. baseline 2.62M to 2.16M after AKC3K2 compression). The net result is a model that is both more accurate and more lightweight—a rare balance for agricultural edge AI. We thus argue that our contribution is not the modules themselves, but the validated design paradigm for fusing lightweight global and adaptive local processing in resource-constrained agri-vision tasks.

Comparative Analysis: A detailed discussion comparing our results with those from key recent studies on coffee fruit maturity detection is shown in **Table 9**, analyzing the differences and the underlying reasons, particularly in light of our algorithmic improvements.

Limitations: The experiments rely on an expanded dataset of 800 images (public data + field captures from a single geographic region) augmented to balance samples. However, the limited absolute size, geographic scope, and cultivar diversity restrict the model’s broad generalizability, with performance unvalidated across other growing regions, distinct lighting, or seasonal conditions. Despite augmentation, natural class imbalance (e.g., more unripe vs. fewer overripe samples) and potential annotation inconsistencies at occluded boundaries may bias decision boundaries and impact per-class performance—aggregate metrics may mask underperformance in specific categories. The SSViT module’s lightweight design (single-scale encoder, 2D positional encoding on the deepest C5 feature map) preserves global context with low overhead but may miss multi-scale global interactions of full multi-layer/multi-scale Transformers, limiting gains in highly heterogeneous scenes. While the model improves partially occluded fruit detection, very severe occlusion, extreme illumination (e.g., direct glare, night scenes), foliage-fruit color similarity, and heavy motion blur remain challenging, leading to potential misses or misclassifications. Additionally, the AKC3K2 module reduces parameters by 40.1% (from 2.62M to 1.57M) but only boosts FPS by 30% (from 20 to 26), indicating parameter count is not the sole determinant of inference latency—hardware-specific execution efficiency may affect practical deployment.

Future work: Expand and diversify the dataset with multi−site, multi−cultivar, multi−season captures and consider additional imaging modalities (multispectral/NIR) to improve model robustness and enable cross−domain validation. Investigate model compression and acceleration techniques (post−training quantization, knowledge distillation, operator fusion) and benchmark on representative embedded platforms to validate real−time applicability. Explore integrating temporal information from short video sequences or multi−view fusion to mitigate occlusion effects and improve detection continuity in harvesting workflows (Zhang et al., 2025). Consider semi−supervised or active learning strategies to reduce labeling costs while extending coverage of rare maturity states.

## 5 Conclusions

In this paper, we present SSViT−YOLOv11, a lightweight detector that combines Arbitrary Kernel Convolution (AKConv/AKC3K2), a Single−Scale Vision Transformer module (SSViT), and Multi−Scale Convolutional Attention (MSCA) in the detection head to address challenges in coffee fruit maturity detection (small targets, occlusion, subtle inter−class visual differences). Comprehensive ablations demonstrate that each proposed component contributes positively and that their combination attains the best overall performance. On the tested dataset SSViT−YOLOv11 achieves precision of 0.811, recall of 0.774, mAP@0.5 of 84.54%, runs at 23 FPS and requires 2.16M parameters—indicating a favorable balance between accuracy, latency and compactness for practical deployment. Compared with several contemporary YOLO and ViT−based detectors, SSViT−YOLOv11 achieves superior or competitive detection accuracy while remaining significantly more parameter−efficient and faster in most settings evaluated. Future work will focus on dataset expansion across cultivars and environments, more rigorous metric reporting, and engineering the model for robust embedded inference in real harvesting systems.

## Data Availability

The original contributions presented in the study are included in the article/supplementary material. Further inquiries can be directed to the corresponding author/s.
